# Promotion of hair growth by a conditioned medium from human umbilical cord mesenchymal stem cells cultivated in a 3D scaffold of gelatin sponge

**DOI:** 10.1186/s40001-024-01830-7

**Published:** 2024-05-04

**Authors:** Jintao Ni, Danyan Ye, Weiping Zeng, Siyi Ma, Zhixia Wang, Yuping Kuang, Lujun Yang

**Affiliations:** 1 Department of Plastic Surgery and Burns Center, Second Affiliated Hospital of Shantou University Medical College, Shantou, Guangdong China; 2 Research Center for Translational Medicine, Second Affiliated Hospital of Shantou University Medical College, Shantou, Guangdong China

**Keywords:** Hair growth, Human umbilical cord mesenchymal stem cell, Conditioned medium, Gelatin sponge, Angiogenesis

## Abstract

**Background:**

This study aims to investigate the effects of a conditioned medium (CM) from human umbilical cord mesenchymal stem cells (HuMSCs) cultivated in gelatin sponge (GS-HuMSCs-CM) on hair growth in a mouse model.

**Methods:**

CM was collected from the HuMSCs cultivated in a monolayer or in a gelatin sponge. Vascular endothelial growth factor (VEGF), insulin-like growth factor-1 (IGF-1), keratinocyte growth factor (KGF), and hepatocyte growth factor (HGF) levels in CMs were measured by enzyme-linked immunosorbent assays (ELISAs). A hair loss model by a C57 BL/6J mouse was prepared. The effects of GS-HuMSCs-CM and HuMSCs on hair regrowth in mice were investigated by intradermal injection in the depilated back skin with normal saline (NS) as the control. The time for hair regrowth and full covering in depilated areas was observed, and the hair growth was evaluated histologically and by grossly measuring hair length and diameter.

**Results:**

Compared with monolayer cultured cells, the three-dimensional (3D) culture of HuMSCs in gelatin sponge drastically increased VEGF, IGF-1, KGF, and HGF production. GS-HuMSCs-CM and HuMSCs injection both promoted hair regeneration in mice, while GS-HuMSCs-CM presented more enhanced effects in hair length, hair diameter, and growth rate. GS-HuMSCs-CM significantly promoted angiogenesis in injected skin areas, which might also contribute to faster hair regrowth.

**Conclusion:**

GS-HuMSCs-CM exerted significant effects on inducing hair growth and promoted skin angiogenesis in C57BL/6J mice.

## Background

Hair loss is typically divided into non-scar alopecia and scar alopecia. Androgenic alopecia (AGA) is the major type of non-scar alopecia, affecting 60–70% of the population [1]. AGA not only affects patients’ appearance but also exerts heavy physical and mental pressure, severely affecting their quality of life and social activity [2]. The hair follicle is a micro-organ that contains epithelial and mesenchymal compartments and is a highly sensitive skin appendage and self-regenerating system, which undergoes life-long cycles, namely rapid growth stage (anagen), regression stage (catagen), and resting stage (telogen) [3]. Alopecia is closely related to the abnormal hair growth cycle, including the shorter duration of the anagen phase, a reduced percentage of anagen phase hair follicles, and the prolongation of the telogen stage [4]. Therefore, the treatment strategies for hair loss include prolonging anagen and reducing the number of hair follicles in telogen. Currently, the treatments for hair loss include drugs such as finasteride and minoxidil, hair transplant, low-level laser therapy, cosmetic camouflages, platelet-rich plasma (PRP), and extracts from cultured MSCs, which presented reliable anti-AGA effects with fewer side effects and with sustainable production and supply [5, 6].

Stem cells, which possess the capacity for self-renewal and multipotent differentiation, have gained significant research attention as a potentially effective therapy. Mesenchymal stem cells (MSCs) are a popular cell source for stem cell-based therapies and are found in bone marrow, umbilical cord blood, Wharton’s jelly, placenta, adipose tissue, and dental pulp tissue [7]. However, MSC therapy possess potential risks including infectious disease, immunological rejection, acute immunogenic responses, and long-term chronic immunogenicity or tumorigenicity [8]. MSCs produce a variety of growth factors that enter the microenvironment and activate neighboring cells [9], and promote tissue repair and regeneration largely through paracrine action by the growth factors [10]. The conditioned medium, which contains the secreted growth factors from cultivated MSCs (MSCs-CM), is considered a potentially effective approach for regenerative medicine [11].

 Human umbilical cord mesenchymal stem cells (HuMSCs) are isolated from Wharton’s jelly. They have broad clinical application prospects in brain, bone, myocardium, lung, pancreas, liver, and autoimmune diseases [12–18]. Compared with other stem cell sources, HuMSCs exhibit a high proliferation rate; short reproduction time; low immunogenicity; high safety; easier isolation, culture, and manipulation; and less genetic mutation during passaging [19]. Collecting HuMSCs is painless and non-invasive; thus, the procedure is robust against ethical controversy [20]. Currently, MSCs, such as adipose-derived stromal cells (ADSCs), bone marrow MSCs (BM-MSCs), and dental-derived mesenchymal stem cells have been analyzed in hair-loss treatment [21–23]. Moreover, the cell-free MSCs-CM could promote hair regeneration [24]. HuMSCs were found to secrete higher concentrations of growth factors and cytokines compared with other MSCs sources [25, 26]. Growth factors and cytokines in HuMSCs-CM potentially modulate the micro-environment and may thereby effect significant roles in hair growth.

The growth factors and cytokines in CM are essential for therapeutic strategies, and several approaches have been developed to increase the levels of growth factors and cytokines secreted by MSCs. Hypoxia benefits the growth factor secretion of ADSCs and promotes hair growth [27]. A low dose of ultraviolet radiation B (UVB) significantly stimulated the paracrine effects of ADSCs and increased the secretion of ADSCs growth factors, thus inducing hair follicle formation and promoting hair regeneration in mice [28]. In our previous study, HuMSCs were seeded in a 3D structure of platelet-poor plasma (PPP) gel and manifested an increased concentration of growth factors and cytokines in CM [26]. We also described an approach for seeding HuMSCs in a poly-L-lysine (PLL)-coated gelatin sponge and demonstrated that the proposed culture consistently secreted significantly higher levels of growth factor compared to monolayer cultures [29].

We propose that the CM collected from HuMSCs cultivated in a gelatin sponge (GS-HuMSCs-CM) might be an effective strategy for promoting hair growth. To verify this hypothesis, we prepared CM from the 3D cultured HuMSCs and investigated the effects on hair growth in C57BL/6 mice in vivo, with HuMSCs and normal saline as contrasts.

## Materials and methods

### Isolation and culture of human umbilical cord mesenchymal stem cells

The study was approved by the Ethics Committee of Shantou University Medical College (SUMC, Shantou, China). Umbilical cords were obtained from healthy pregnant women (excluding those with HIV, hepatitis, and syphilis) at term (38–40 weeks) through cesarean section in the Obstetrics and Gynaecology Department of the Second Affiliated Hospital of SUMC, after obtaining written informed consent. HuMSCs were prepared as previously described [26]. The umbilical cord was cut into 2–3 cm pieces with a sterile scalpel and cleaned with sterile phosphate-buffered saline (PBS). After removing the umbilical arteries, veins, and umbilical cord adventitia from the umbilical cord, Wharton’s jelly was obtained. Wharton's Jelly tissues were cut into 2 mm^2^ pieces and attached to the bottom of 100 mm cell culture dishes (JET BIOFIL, China). The culture dishes were subsequently inverted at 5% CO_2_ and 37 ℃ for 15–30 min until tissue blocks adhered to the bottom of the dishes. Dulbecco’s High Glucose Modified Eagle’s Medium (DMEM, Gibco; Thermo Fisher Scientific, Inc., Waltham, MA, USA) containing 2% fetal bovine serum (Gibco, USA), 1% penicillin–streptomycin (Beyotime, Shanghai, China), transferrin (Sigma-Aldrich Co., USA), basic fibroblast growth factor (bFGF, Zhuhai Essex Pharmaceutical Co., LTD., China), insulin (Sigma-Aldrich, USA), and selenium acid (Aldrich, Sigma-Aldrich, Co., USA), as previously described [30], was added, and the tissue blocks were incubated in a humidified CO_2_ incubator at 37 °C. The medium was replaced after HuMSCs grew out of the tissues after 5–7 days.

### Conditioned medium from human umbilical cord mesenchymal stem cells seeded in gelatin sponge

The conditioned medium of HuMSCs used was the 3rd-5th passage. GS-HuMSCs-CM was prepared as previously described [29]. Briefly, gelatin sponges (Xiang’en, Jiangxi Medical Technology Development Co., LTD., China) were cut into 20 mm × 20 mm × 10 mm pieces in a sterile environment. Subsequently, gelatin sponges were coated with PLL (Beyotime, China). The third-generation HuMSCs were seeded in PLL-coated gelatin sponges (GS-HuMSCs). Each gelatin sponge piece was seeded with 1.5 mL HuMSCs suspension (3 × 10^6^ cells /mL) and incubated in a 6-well plate at 5% CO_2_, 37 ℃ for 30 min. GS-HuMSCs were cultured in a 6 mL culture medium with 1 μL/mL ascorbic acid at 37 °C with 5% CO_2_ for 48 h. Conditioned medium was harvested from three groups: a group of HuMSCs at 100% confluency, a group of monolayer culture HuMSCs (Day 3 after confluency), and a group of GS-HuMSCs-CM (Day 3 after seeded in gelatin sponge). One day before the conditioned medium collection, the culture medium was changed to be serum-free. The CM was collected and centrifuged at 3000 rpm for 10 min to remove floating cells and cell debris, and then allotted and preserved at −80 °C prior to ELISA analysis. The cells were harvested and counted after CM collection.

### Evaluation of growth factor levels (ELISA assay)

The CM of HuMSCs at 100% confluence, HuMSCs-CM (Day 3), and GS-HuMSCs-CM (Day 3) were collected, and the levels of VEGF, IGF-1, KGF, and HGF in CM were quantified by ELISA kits (R&D Systems, USA) according to the manufacturer’s instructions. All data were compiled from a minimum of three replicate experiments, with each performed in duplicate, and growth factor levels were calculated from four-parameter logistic curves. Finally, the growth factor concentration was adjusted to be ng (or pg) /ml/million cells.

### Animals and ethical approval

6–7 week-old wild-type male C57BL/6J mice, weighing 20–22 g, were purchased from the Animal Center of Shantou University Medical College (Shantou, China). All protocols were approved by the Ethics Committee of the SUMC, and all animal experiments were approved by the Institutional Animal Care and Use Committee of the Shantou University Medical College and conducted according to the guidelines of the National Health and Medical Research Council (China).

### Animal experiments

**Fig. 1:**
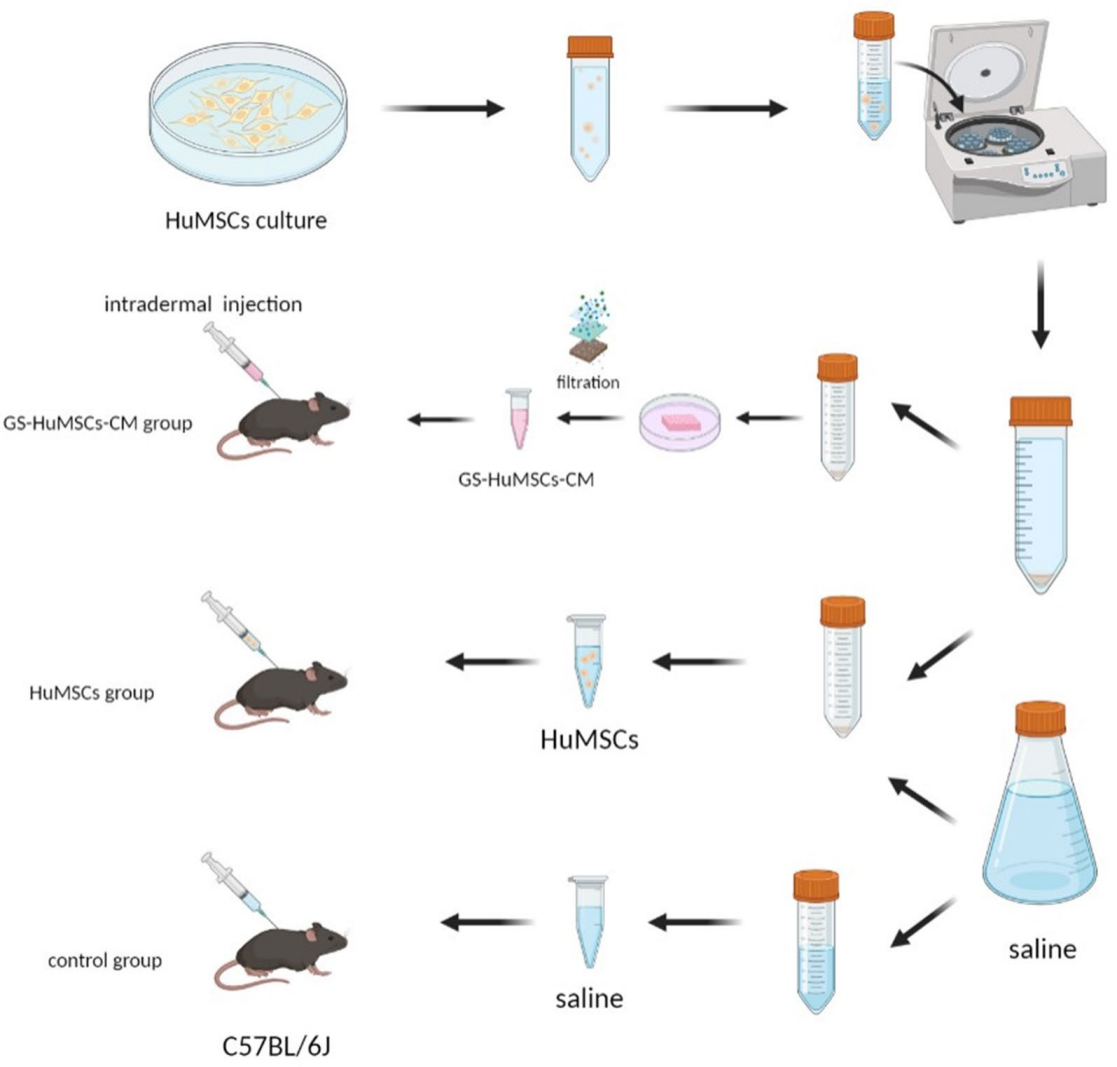
Schematic diagram of the preparation of HuMSCs, GS-HuMSCs-CM, experimental model and treatment process. Saline (control), HuMSCs, GS-HuMSCs-CM, respectively, were injected subcutaneously in the dorsal skin of each mouse once per 3 days for three times. *HuMSCs* Human umbilical cord mesenchymal stem cells, *GS* gelatin sponge, *CM* conditioned medium

The mice were maintained in a 12 h light–dark cycle under a room temperature of 26 ± 2 °C. The C57BL/6J male mice were kept for one week before the experiments. The mice were anesthetized by inhaling isoflurane. The dorsal hair (2 cm × 4 cm) was shaved with an electric clipper and completely removed using hair remover cream (Reckitt Benckiser France, CEDEX, France). The mice were randomly assigned to three groups (n = 9 each): the control group (normal saline), HuMSCs group, and GS-HuMSCs-CM group. Mice in the control group were administered normal saline, mice in the HuMSCs group were administered 1 × 10^7^ fresh HuMSCs suspended in normal saline, and mice in the GS-HuMSCs-CM group were administered GS-HuMSCs-CM, 16 points for intradermal injection, and 100 uL/point, once per three days for three times. Intradermal injections were administered with a 31-gauge needle at a depth of approximately 1 mm. (Fig. 1).

### Hair growth observation

Skin darkening and hair growth were monitored. The hair growth score was evaluated as described by Vegesna et al. [31]. Briefly, a score of 0 indicated that the dorsal skin is still pink compared with the start time of the hair growth induction, and a score of 10 indicated full hair regrowth in the entire depilated area. The morphological changes of each group injected with normal saline (control), HuMSCs, or GS-HuMSCs-CM at day 0, day 7, day 10, day 14, and day 21 were captured by a digital camera (EOS 6D, Canon, Japan). The hairs of three areas (1 cm^2^) on the back of mice on day 14 and day 21 were obtained and weighed on a Sartorius BSA224S-CW 1/10,000 analytical balance (Sartorius, Beijing, China). On day 14 and day 21, thirty hairs were randomly selected from the same area of each mouse, the longest and the shortest hairs were excluded, and ten hairs were retained for length and thickness measurement using a micrometer (SangNond, Shenzhen, China) with a 0.01 mm at 10 × magnification accuracy under an inverted microscope (Olympus CKX31).

### Hematoxylin and eosin (H&E) staining

On day 7, day 14, and day 21 after treatment, three mice were randomly selected from each treatment group and sacrificed. Skin samples were harvested, fixed, embedded in paraffin, and sectioned at 4 µm. Sections were rehydrated, dewaxed, and stained with hematoxylin–eosin (H&E) according to respective protocols. The images were captured and viewed using a microscope at 40 × and 400 × magnification. The hair-growth conditions and the hair follicle’s growth phase were observed under the microscope. The hair follicles in six randomly selected fields of view (200 ×) were counted under a microscope, and the average was calculated.

### Statistical analysis

Each experiment was repeated at least three times. The data were statistically analyzed using one-way ANOVA and the least-squares difference test for the comparison among groups using SPSS ver. 25.0 (SPSS Inc., Chicago, IL, USA) for Windows statistical package. Significance was defined as *P* < 0.05. All data were reported as a mean ± standard error of the mean (SEM) of at least 3 separate experiments.

## Results

### Morphology of umbilical cord mesenchymal stem cells

**Fig. 2:**
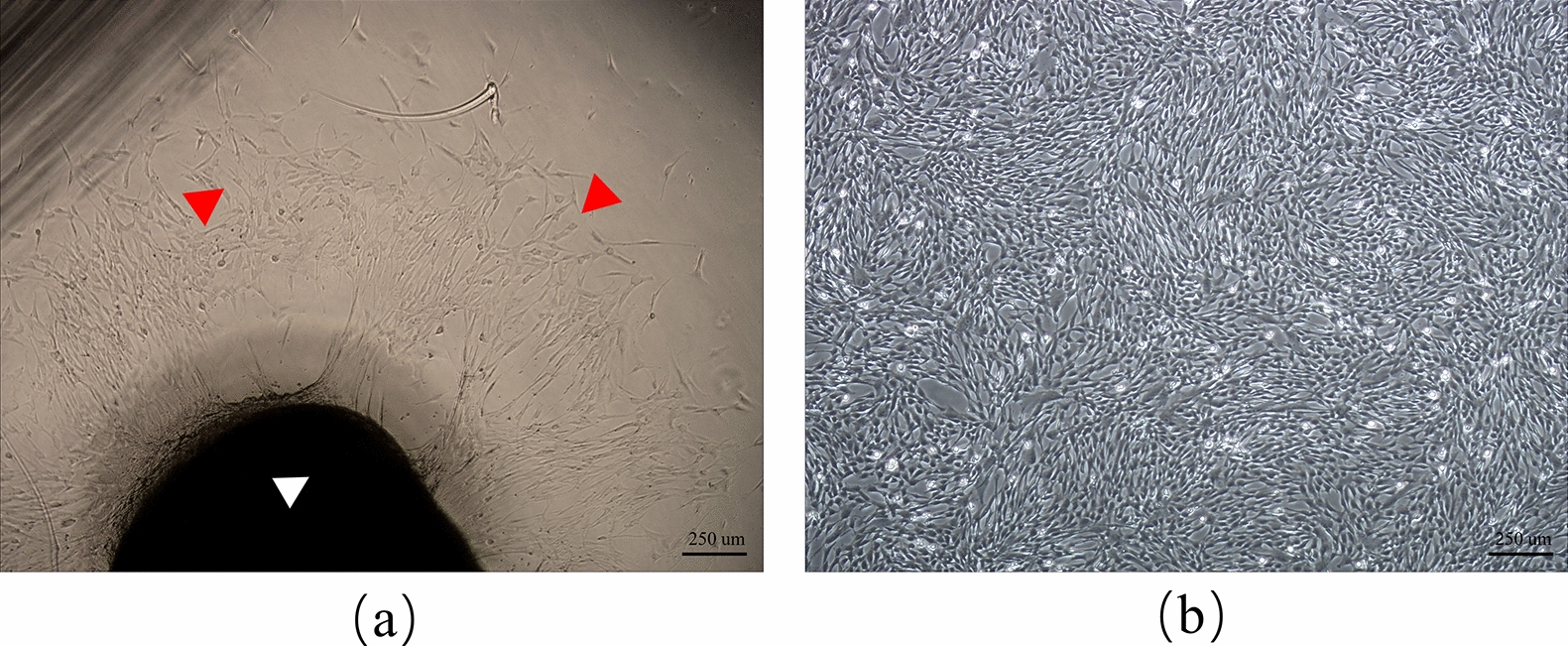
Morphology of primary and passage cultured HuMSCs. **a** Primary cultured HuMSCs at Day 7. The HuMSCs (red triangle) migrated out from adherent tissue Wharton's jelly tissues (white triangle). HuMSCs showed fibroblast-like morphology. **b** The HuMSCs were inoculated into the petri dishes at a concentration of 1.5 × 10^5^. **c** HuMSCs reached 80–90% confluence. *HuMSCs* human umbilical cord mesenchymal stem cells

Around the edges of the block tissues, cells started growing, which was observed under the inverted microscope (Olympus CKX31) (Fig. 2a). Cells were passaged till their proliferation attained 80–90% confluency (Fig. 2b).

### Growth factor levels increased in GS-HuMSCs-CM

**Fig. 3:**
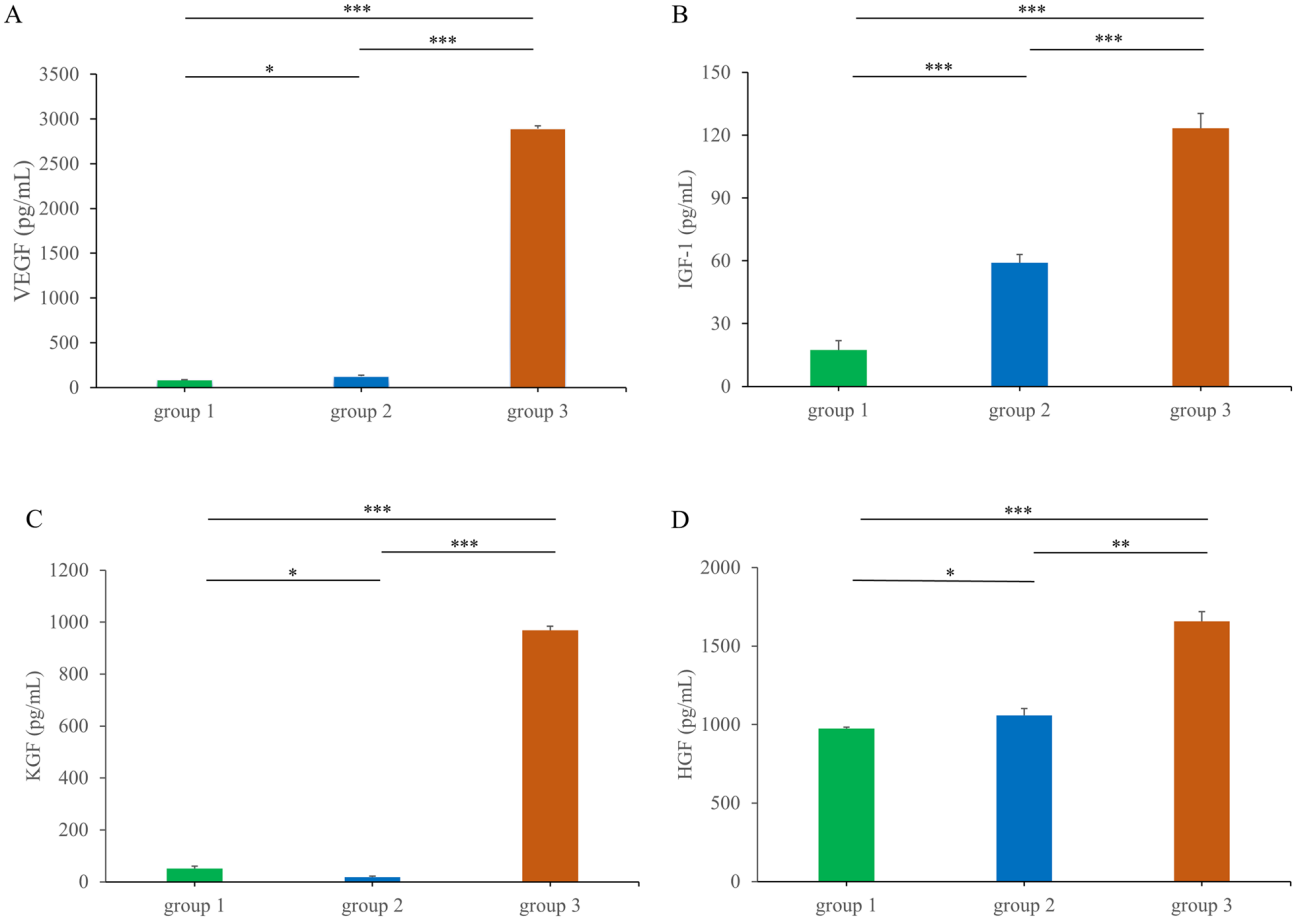
Growth factor assessment of HuMSCs100% confluence group, monolayer group, and GS-HuMSCs-CM groups. The conditional medium of HuMSCs100% confluence was collected, and the conditional medium of monolayer group and GS-HuMSCs-CM group was collected on the third day. **A**–**D** showed the secretion trend of VEGF, IGF-1, KGF and HGF. (*p > 0.05, **p < 0.01, ***p < 0.001). Group 1: 100% confluence group; Group 2: monolayer group (day 3); Group 3: GS-HuMSCs-CM group

We investigated the growth factors in CMs. The levels of VEGF, KGF, HGF, and IGF-1were all markedly higher in the GS-HuMSCs-CM group than in the monolayer culture HuMSCs-CM (Day 3 after confluency) and HuMSCs100% confluence groups (Fig. 3).

### Hair-growth-promoting effects of GS-HuMSCs-CM, HuMSCs, and saline in C57BL/6J mice

It is generally accepted that the dorsal hair of the C57BL/6J mouse exhibits a time-synchronized hair-growth cycle. The depilated skin of mice was pink in telogen. After the intradermal injection of saline (control), HuMSCs, and GS-HuMSCs-CM on the back of mice three times, we observed the changes in skin color and hair growth.

**Fig. 4:**
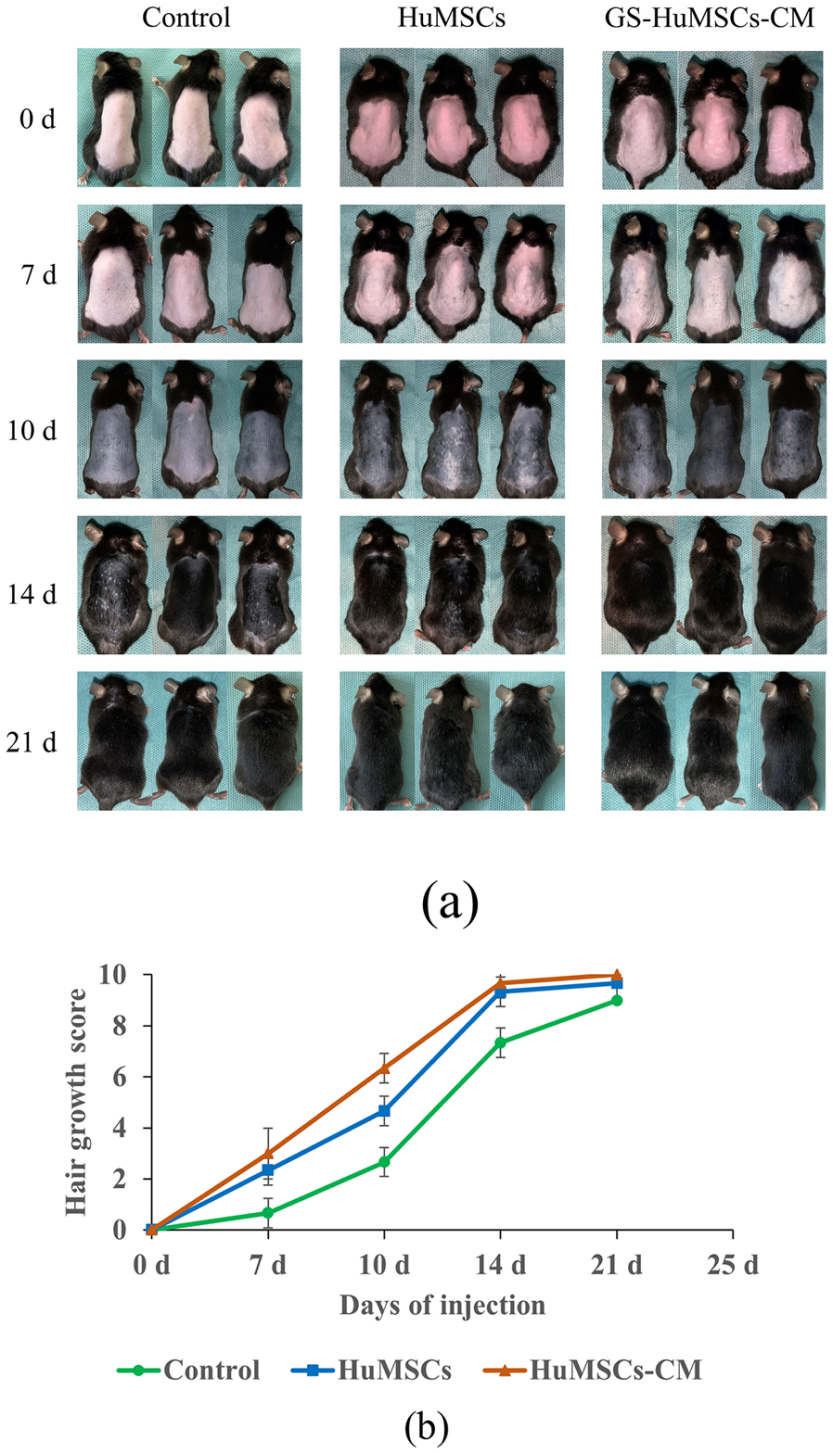
Hair growth effect of GS-HuMSCs-CM in hair loss induced C57BL/6 J mice. The hairs were depilated from the backs of C57BL/6 J, and the hair growth rate was observed 3 times per week for 21 days. The backs of mice were injected with GS-HuMScs-CM, HuMSCs, and saline (control), respectively, once per 3 days for 3 times. The gross view was observed by photographs (**a**), and hair growth scoring was measured (**b**). From day 5 to day 15, mice in the GS-HuMSCs-CM injection group showed significantly increased hair growth compared with the HuMSCs group (**P < 0.05, n = 9). The GS-HuMSCs-CM group and the HuMSCs group showed significantly increased hair growth compared with the control group (^#^*P* < 0.01, n = 9; ∗*P* < 0.05, n = 9)

Mice injected with GS-HuMSCs-CM exhibited more rapid hair growth than mice injected with HuMSCs and saline (Fig. 4a, b). On day 7 after injection, mice in the GS-HuMSCs-CM and HuMSCs treatment groups manifested marked dark spots on depilated skin, while there were no marked spots in the control-group mice. Hair shafts of the GS-HuMSCs-CM and HuMSCs treatment groups were visible on day 10 after injection. Specifically, the skin of the GS-HuMSCs-CM group was darker than that of the control group on day 10 after depilation. More hair shafts were observed in GS-HuMSCs-CM groups than in HuMSCs and the control group (Fig. 4). On day 14, 95%–100% of hair coverage was observed in the GS-HuMSCs-CM group, 85%–90% of hair coverage in the HuMSCs group, and 40%–70% of hair coverage in the control group. On day 21, the hair was overgrown in the depilated area on the back of the GS-HuMSCs-CM group and HuMSCs group, but not in the control group.

**Fig. 5:**
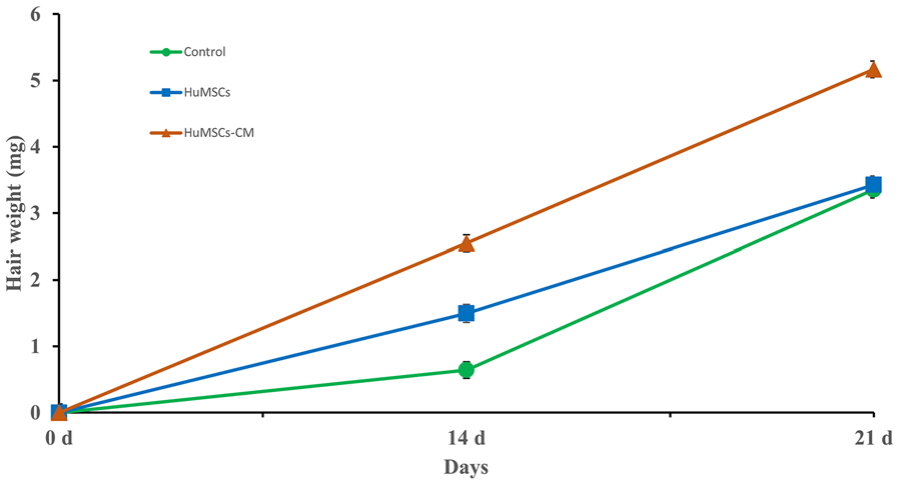
Hair weight at Day 14and Day 21after injection of GS-HuMSCs-CM, HuMSCs, and saline. The hair weight of mice in GS-HuMSCs-CM group and HuMSCs group were significantly weighter compared with the control group (**p < 0.01, *p < 0.05, n = 6). The HuMSCs injection group also showed significantly weight hair regeneration compared with the control group (∗*p* < 0.05, n = 6)

**Fig. 6:**
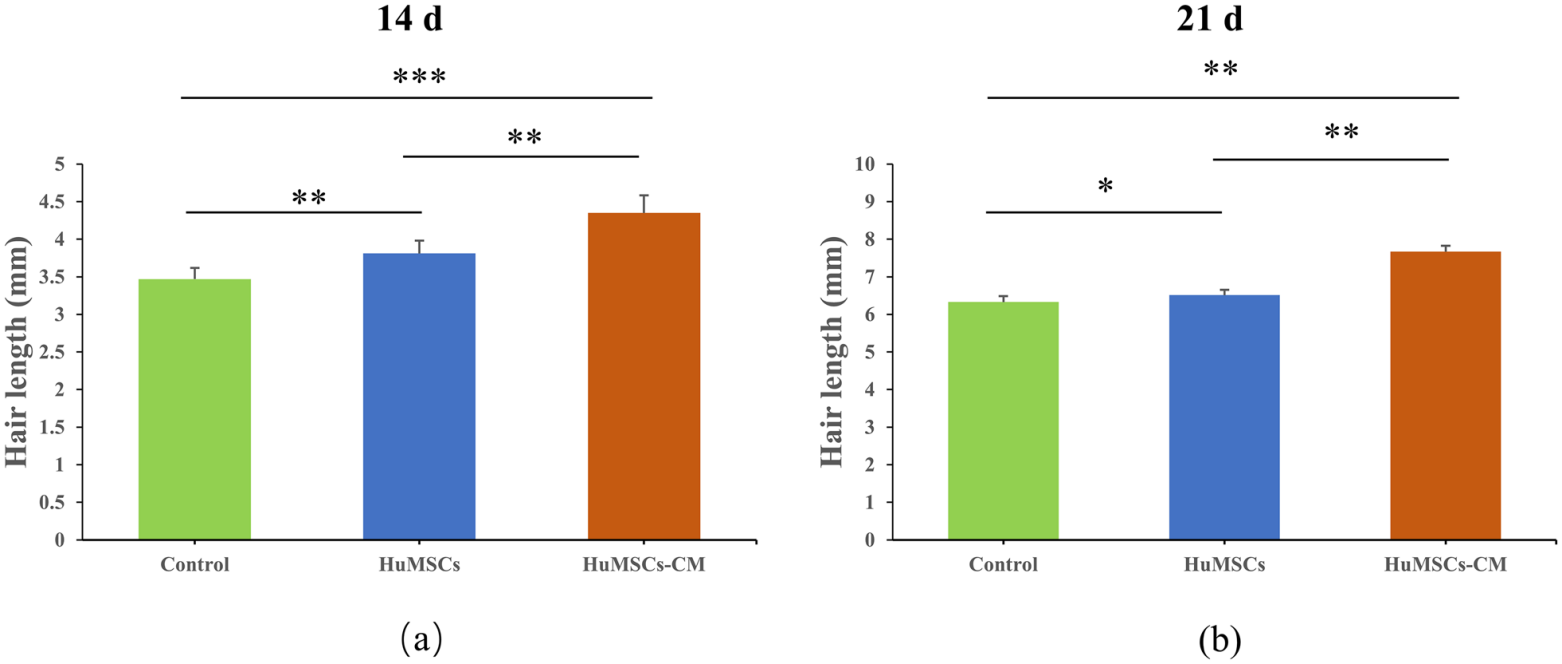
Hair growth effect of GS-HuMSCs-CM in hair loss induced C57BL/6 J mice. Hair length at Day 14, Day 21 after subcutaneous injection of saline (control), HuMSCs and GS-HuMSCs-CM. Hair length was measured for Day 14 (**a**), Day 21 (**b**). Mice in the GS-HuMSCs-CM injection group showed significantly increased hair length compared with the HuMSCs injection group (**p < 0.01, n = 10) and negative control group (***p < 0.001, n = 10). The HuMSCs injection group also showed significantly increased hair length compared with the control group (**p < 0.01, *p < 0.05, n = 10). The data shown represent one of each group, and experiments were performed three times

**Fig. 7:**
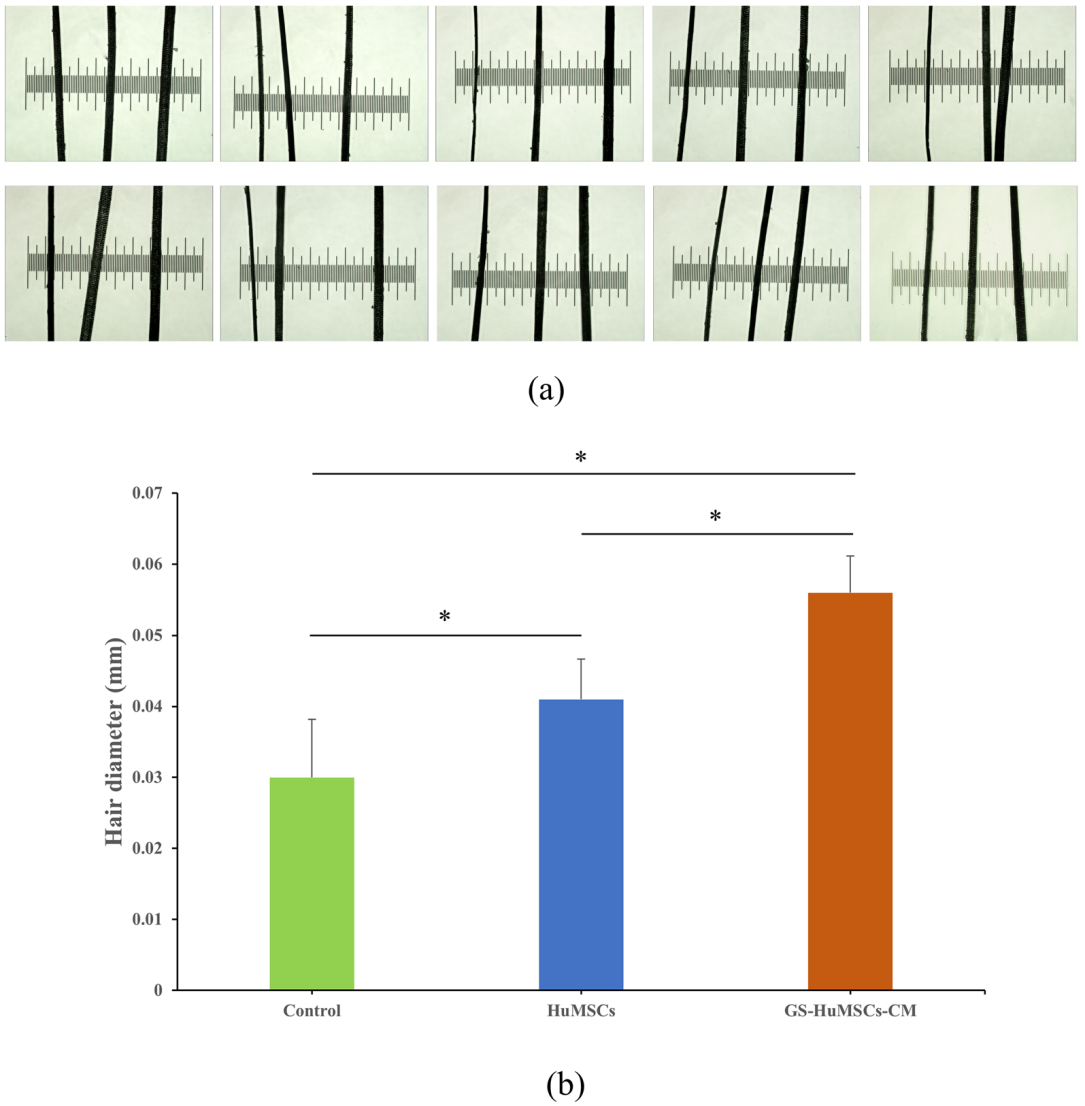
Hair diameter at Day14 after injection of GS-HuMSCs-CM, HuMSCs, and saline. The diameter of the hair in the GS-HuMSCs-CM group, HuMSCs group and the control group were measured by microscopically side microscale and they were compared (**a**). The hair diameter of mice in GS-HuMSCs-CM group and HuMSCs group were significantly thicker compared with the control group (*p < 0.01, n = 10). The HuMSCs injection group also showed significantly weight hair regeneration compared with the control group (∗*p* < 0.01, n = 10) (**b**)

Hair was collected from injected areas on day 14 and day 21 of each group. On day 14 and day 21 after the injection, the hair weight in the GS-HuMSCs-CM group (2.55 ± 0.13 mg and 5.17 ± 0.04 mg, respectively, n = 6) was significantly heavier than that in the HuMSCs group (1.49 ± 0.03 mg and 3.42 ± 0.03 mg, respectively, n = 6) and control group (0.64 ± 0.01 mg and 3.35 ± 0.02 mg, **P < 0.01; n = 6). On day 14, the HuMSCs group (1.49 ± 0.03 mg, respectively, n = 6) was heavier than the control group (0.64 ± 0.01 mg, **P < 0.01, n = 6). However, on day 21, there was no statistical difference between the HuMSCs group and control group (*P > 0.05) (Fig. 5). On day 14 and day 21 after the last injection, hair length measurements were performed. On day 14 (Fig. 6a) and day 21 (Fig. 6b) after the last injection, the hair length of mice in the GS-HuMSCs-CM group (4.35 ± 0.23 and 7.67 ± 0.16 mm, respectively, n = 10) was significantly longer than that in the HuMSCs group (3.81 ± 0.17 and 6.55 ± 0.14 mm, **P < 0.01, respectively, n = 10) and control group (3.47 ± 0.15 and 6.33 ± 0.16 mm, ***P < 0.001, respectively, n = 10). The hair length of mice in the HuMSCs group (3.81 ± 0.17 and 6.55 ± 0.14 mm, respectively, n = 10) was longer than that of mice in the control group (3.47 ± 0.15 and 6.33 ± 0.16 mm, **P < 0.01, *P < 0.05, n = 10) (Fig. 6). On day 14 after the last injection, the diameters of 10 hairs were measured under the microscope with a microscale (Fig. 7a). The hair diameter of mice in the GS-HuMSCs-CM group (0.056 ± 0.005 mm, n = 10) was significantly thicker than that of mice in the HuMSCs group (0.041 ± 0.006 mm, *P < 0.01, n = 10) and control group (0.030 ± 0.008 mm, *P < 0.01, n = 10). The hair diameter of mice in the HuMSCs group (0.041 ± 0.006 mm, *P < 0.01, respectively, n = 10) was thicker compared with the control group (0.030 ± 0.008 mm, *P < 0.01, respectively, n = 10) (Fig. 7b).

### GS-HuMSCs-CM increased neo-angiogenesis in C57BL/6 Mice

**Fig. 8:**
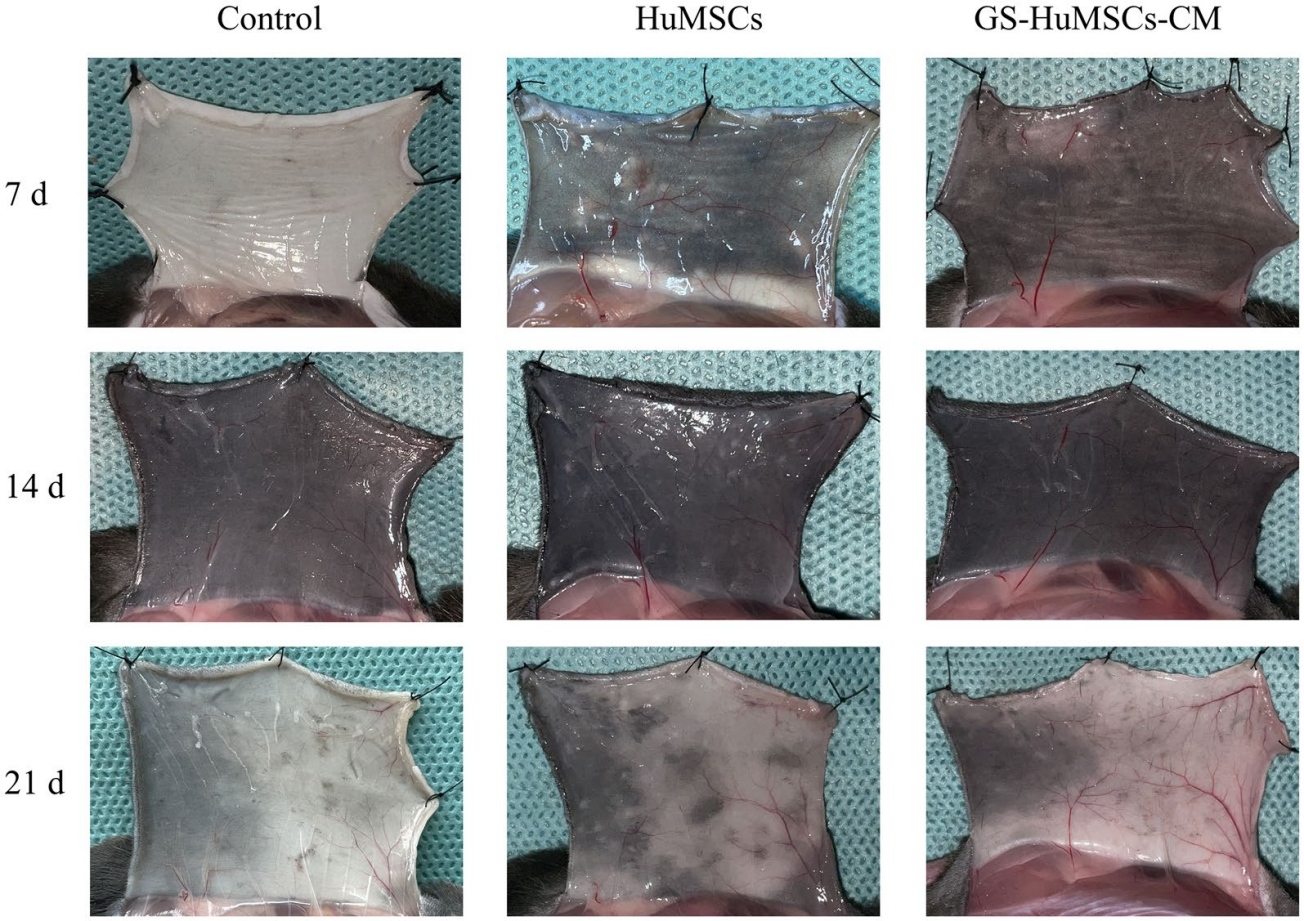
Stimulatory effect of GS-HuMSCs-CM on angiogenesis. The vascularization of the hair regeneration site was analyzed Day 7, Day 14 and Day 21 after injection. The skin of the hair regeneration site in the GS-HuMSCs-CM group and the HuMSCs group showed mature vascular branches. The control group had smaller vessels with fewer branches. Mature angiogenesis was significantly enhanced in the GS-HuMSCs-CM group

Mice were sacrificed, and the back skin was turned over on day 7, day 14, and day 21 after the last injection. Compared with the control group, blood vessels increased in number and diameter in the GS-HuMSCs-CM group and HuMSCs group on day 7. However, no apparent changes were noted in the control. On day 14 and day 21, the GS-HuMSCs-CM group and HuMSCs group manifested higher blood vessel density and thicker and more branching vessels in the dorsal skin. However, the dorsal vessels of the control group were smaller in diameter, with lower density and sparser branches. The number of neoangiogenesis in the GS-HuMSCs-CM group was higher than that in the HuMSCs group on days 7, 14, and 21 (Fig. 8).

### Histological findings of dorsal hair follicle changes in mice

The growth cycle of hair follicles was histologically observed according to a protocol described by Muller et al. [32]. Skin tissue was harvested and processed for histological investigation.

**Fig. 9:**
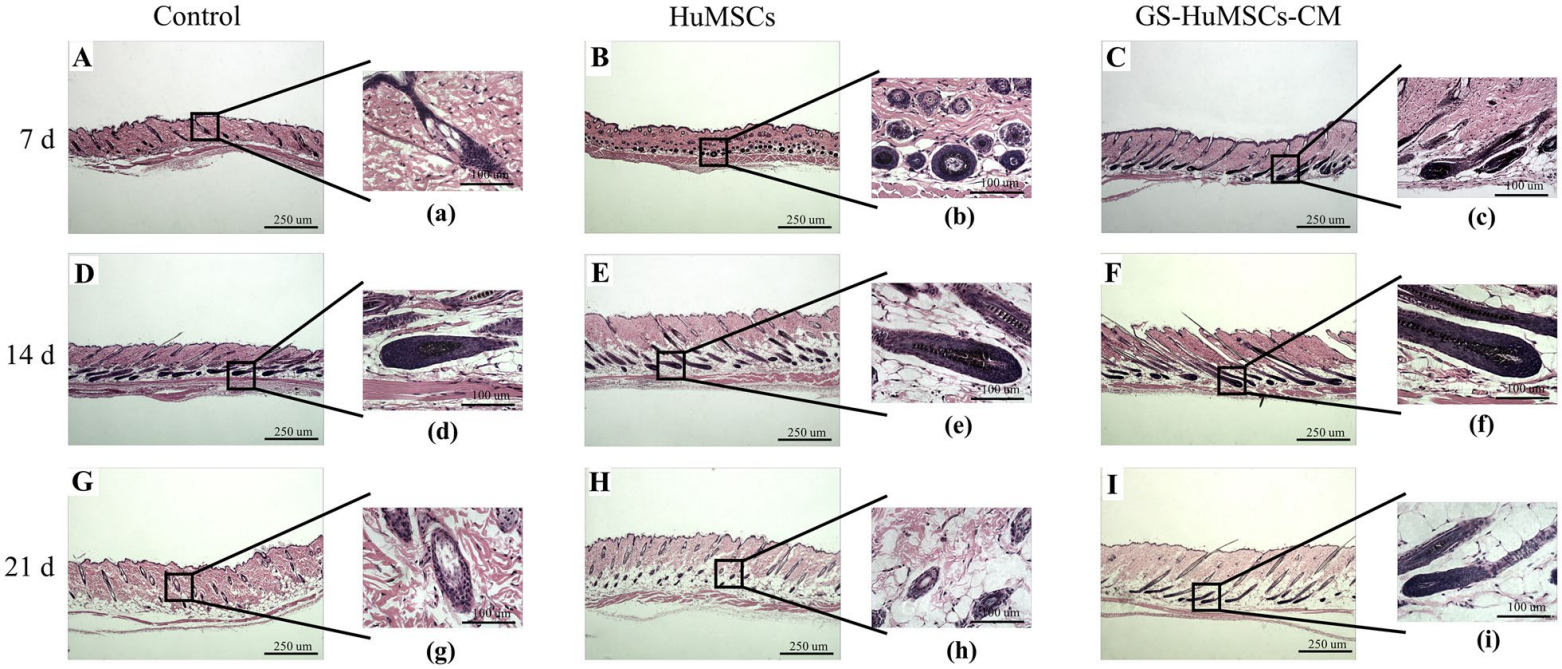
GS-HuMSCs-CM promotes the conversion from telogen to anagen in C57BL/6 J mice. Histological evaluation of the control group, HuMSCs group and GS-HuMSCs-CM group (Day 7, Day 14 and Day 21). **A**–**I** is viewed under 40 × microscope field. a–i is viewed under 400 × microscope field. **A** (a)–**C** (c) shows the changes of hair follicles at Day 7. **D** (d)–**F** (f) shows the changes of hair follicles at Day 14. **G** (g)–**I** (i) showed the change of hair follicles on Day 21. Day 7, the GS-HuMSCs-CM group had entered the Anagen IIIc (c), and the HuMSCs group also entered the Anagen II (b), while the negative group was still in the telogen phase (a). Day 14, the GS-HuMSCs-CM group was still in the Anagen V (f), the HuMSCs group was also in the anagen IIIc (e), and the control group entered the Anagen IIIa (d). Day 21, the GS-HuMSCs-CM group was in the early catagen phase (i), while both the HuMSCs group and the control group entered the telogen phase (h–i)

On day 7, hairs in the GS-HuMSCs-CM group were in the anagen phase (Anagen II), and in Anagen I in the HuMSCs group, while the control group hairs were in the telogen phase. Meanwhile, in the GS-HuMSCs-CM group, the dermis became thicker with larger hair follicles deeper into the subcutaneous tissue than the HuMSCs group, and more melanin was around the dermal papilla. However, the control group’s dermis was thin with hair follicles still residing in the dermal layer. (Fig. 9A (a)—C (c)). On day 14, hairs in the GS-HuMSCs-CM group were in Anagen V, with increased hair number and larger dermal papilla in the hair follicles. Melanin significantly increased, and the inner root sheath (IRS) grew from the dermal papilla and into the dermis. In the HuMSCs group, hairs were in Anagen IIIc, with larger dermal papilla, and melanin increased. The control group entered Anagen IIIa, with a few hair follicles growing, and melanin surrounded the dermal papilla (Fig. 9D (d)—F (f)). On day 21, in the GS-HuMSCs-CM group, the dermal papilla became smaller, the melanin gradually decreased, the hair follicle bulb became narrower, and the hair follicles began to enter early catagen (Catagen II). In the HuMSCs group, the density of hair follicles and melanin decreased, and the hair follicles entered the anaphase stage of catagen (Catagen VI). In the control group, the dermal papilla was separated from the hair shaft, the melanin decreased, and the hair follicles were in catagen (Catagen VIII) (Fig. 9G (g)—I (i)).

**Fig. 10:**
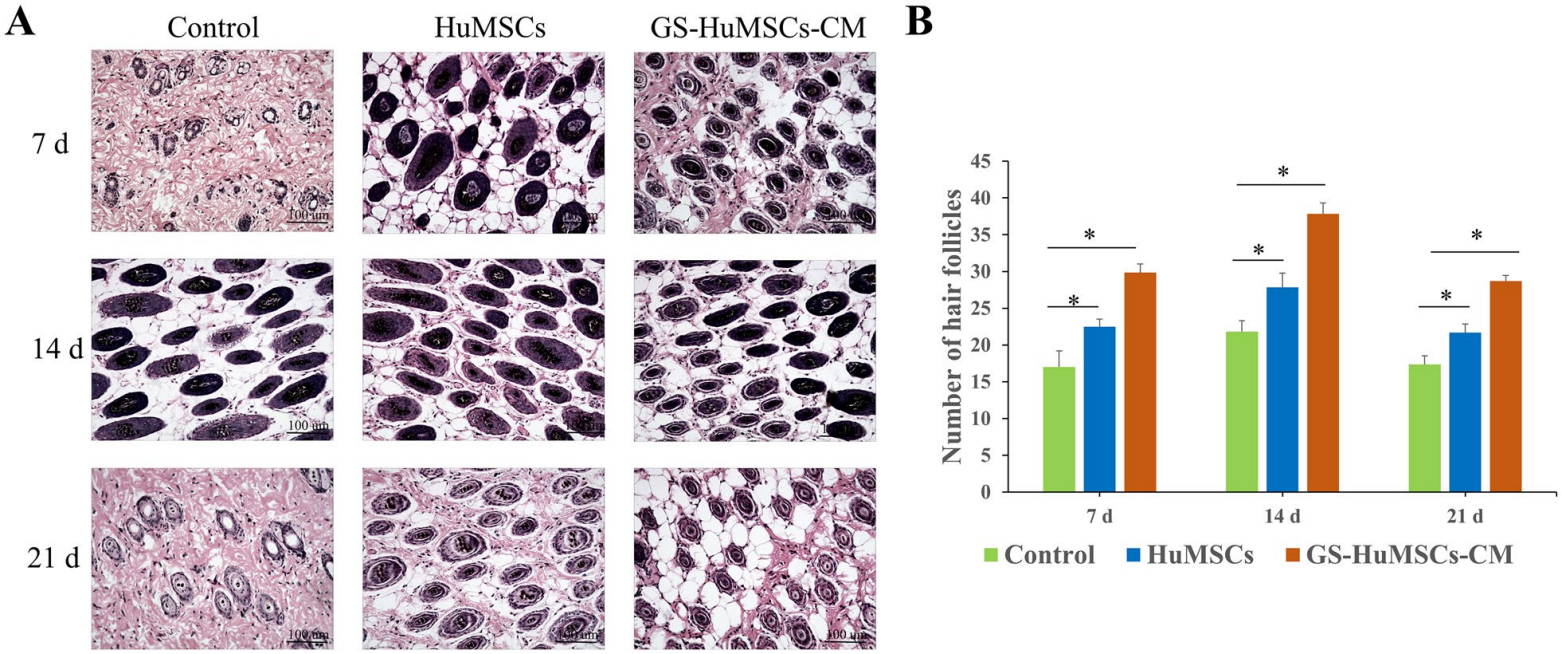
Increased hair growth and hair follicle density in mice by GS-HuMSCs-CM. **A**. Representative hematoxylin–eosin (H&E) images of dorsal skin sections for determination of hair follicle (HF) number. **B**. The number of hair follicles in 6 areas was randomly observed and counted under 200 × microscope field. Data are represented as the mean ± SD (n = 6, *p < 0.01)

On day 7, day 14, and day 21, dorsal skin tissues were harvested and quantified for hair follicle density under a microscope (Fig. 10A). Depilated skin treated with GS-HuMSCs-CM and HuMSCs exhibited higher hair follicle density and more terminal hairs than the tissue treated with saline, and the hair-promoting effect of GS-HuMSCs-CM was stronger than that of HuMSCs (*P < 0.01) (Fig. 10B).

## Discussion

We investigated the ability of HuMSCs secretome to promote hair growth. In an animal model, HuMSCs cultured in a gelatin sponge secreted growth factors and cytokines of high levels into CM, which promoted hair regeneration. GS-HuMSCs-CM exerted hair-growth promoting effects via anagen induction, thereby promoting hair follicle growth and neo-angiogenesis.

The C57BL/6 mouse is the best animal model for hair-cycle analysis. Under favorable feeding conditions, all hair follicles of C57BL/6 mice entered telogen at 6–8 weeks after birth, and in the physiological state, hair follicles no longer spontaneously enter anagen [33]. After traumatic stimulation such as manual depilation or hair pluck, the hair follicles can be induced to enter anagen and then enter telogen after catagen. This cycle of activity is indistinguishable from the natural cycle [34]. This animal model had been successfully utilized in the analysis of hair-loss management [35, 36].

HuMSCs have been widely analyzed in the stem-cell therapy domain owing to their high self-renewal ability, differentiation ability, immune regulation, paracrine effects, anti-fibrotic activity, anti-inflammatory effect, and low ethical controversy [37]. MSCs repair damaged tissues and promote tissue regeneration largely through the paracrine function [38]. Based on the paracrine role of MSCs, several studies have investigated CM as an alternative therapy [39]. Local supplementation of growth factors and cytokines was conducive to hair growth [40]. Growth factors and cytokines extracted from ADSCs, such as VEGF, b-FGF, PDGF, and KGF, positively promote the proliferation of dermal papilla cells and hair growth [40]. Therefore, we speculate that growth factors and cytokines in GS-HuMSCs-CM may be an effective treatment for alopecia. VEGF is involved in the formation of new blood vessels during anagen and stimulates the growth of hair follicles and hair shafts [41]. IGF-1 exerts a crucial role in the regulation of cell differentiation and tissue regeneration during hair follicle development [42]. KGF is an effective cell-protective growth factor that prevents hair follicle apoptosis through the denovo synthesis of proteins [43]. HGF contributes to anagen maintenance by retarding hair follicle regression [44].

Optimizing stem cell culture conditions to up-regulate key growth factors/cytokines for regeneration therapy is a significant strategy. The cultivation of MSCs in 3D scaffolds or in a hypoxic condition can upregulate the paracrine activity of MSCs [26, 27]. Herein, the CM of HuMSCs at 100% confluence, HuMSCs-CM Day 3 after confluency, and GS-HuMSCs-CM (Day 3) were collected. ELISA analysis revealed that VEGF, IGF-1, KGF and HGF were significantly up-regulated in GS-HuMSCs-CM (*P < 0.05) (Fig. 3). As was previously demonstrated, the gelatin sponge provides the cells with a three-dimensional scaffold that is more consistent with the environment in vivo and helps maintain optimal cell survival and secretion efficacy [29]. As a commonly utilized clinical material, gelatin sponge is cheap and easy to obtain and can be used as a cell scaffold for more optimal clinical translation.

The hair follicle cycle consists of three phases: anagen, catagen, and telogen [45]. Hair regeneration can be promoted by prolonging the anagen period, shortening the telogen period, or facilitating the telogen–anagen transition [46]. Growth factors and cytokines, such as from the ADSC-CM, could promote hair growth by inducing the hair cycle’s anagen phase [40]. To induce a synchronized hair cycle, the hairs of dorsal skin were to be depilated [30]. The depilated skin of mice was pink during telogen and darkened at the initiation of anagen. Physical and chemical depilation may induce the C57BL/6J mouse strain to enter anagen; therefore, the time of skin color changes in the mice’s depilation area can be regarded as the hair-follicle time from telogen to anagen. The experimental results indicated that compared with the control group, GS-HuMSCs-CM and HuMSCs could significantly shorten the time of skin color changes and that of hair growth. The histomorphometric analysis indicated that on day 7 after injection, most of the hair follicles were in Anagen in the GS-HuMSCs-CM group and HuMSCs group, and that in the control group, they were still in Telogen. On day 21, hair follicles in the GS-HuMSCs-CM group entered catagen II, and the hair follicles in HuMSCs group entered catagen VI, whereas the hair follicles in the control group entered catagen VIII. The results indicated that GS-HuMSCs-CM and HuMSCs could induce the hair follicles to enter the anagen stage earlier and maintain hair growth for a longer time. The effect of the GS-HuMSCs-CM group was more significant than that of the HuMSCs group.

We found that after days 14 and 21 of the last injection, the hair length in the GS-HuMSCs-CM group and HuMSCs group were longer than that in the control group (*P* < 0.05) and that the hair weight in the GS-HuMSCs-CM group was significantly heavier than that in the control group (*P* < 0.01). However, no significant difference in hair weight was found between the HuMSCs group and the control group on day 21 (*P* > 0.05). The hair diameter in the GS-HuMSCs-CM group and HuMSCs group were thicker than in the control group (*P* < 0.01). GS-HuMSCs-CM and HuMSCs have a positive effect on hair growth by elongating the hair shaft and increasing the hair diameter. NS injection also induced the hair growth cycle from telogen to anagen, which might have occurred through the needle mechanical stimulation.

Hair growth requires adequate nutrition, oxygen, and sufficient blood supply. Angiogenesis around hair follicles is associated with hair cycles [47]. Hair growth is usually accompanied by increased angiogenesis [48]. VEGF is also an effective angiogenic agent for promoting hair growth and increasing the size and number of hair follicles. The effect of growth factors on hair growth is related to the stimulation of angiogenesis [35, 49]. We noted significant dilation of the blood vessels and the increased connections between different blood vessel plexus on the back skin in GS-HuMSCs-CM treated mice. Angiogenesis induced by GS-HuMSCs-CM might also contribute to the anagen phase induction and hair growth.

## Conclusions

In conclusion, GS-HuMSCs-CM promoted hair growth, potentially by inducing and prolongating the anagen phase, and by enhancing skin angiogenesis in the mice model. GS-HuMSCs-CM presented more optimal results than in HuMSCs in promoting hair growth.

### Future perspective

Hair regeneration techniques include the subcutaneous injection of platelet concentrate, stem cells, and conditioned medium. Local injection of concentrated platelet products can provide nutrients for hair, which can promote the telogen period of hair to re-enter the anagen period. The application of stem-cell-related technology to treatment is a prominent research topic in the medical field. Stem cells exhibit unique and powerful differentiation, immunomodulatory, and anti-inflammatory capabilities. Conditioned mediums of MSCs contain a variety of growth factors that can enhance scalp blood supply and hair follicle cycle, exert multiple effects on hair-loss treatment, and have unlimited potential in promoting hair growth and regeneration. The conditioned mediums can avoid some of the risks associated with stem cells. Meanwhile, the conditioned mediums can be prepared into freeze-dried powder, which exhibits certain advantages in use, storage, and transportation, and is a potentially novel treatment method. There are more components in the conditioned medium of HuMSCs. We can determine which protein substances promote hair growth and the expression of related markers in the cell-signaling pathway. Currently, isolated hair follicles during hair transplant surgery are temporarily stored in lactate Ringer's solution at 4 ℃. We can analyze the activity and survival of the isolated hair follicles stored in the conditioned medium of HuMSCs with lactate Ringer's solution and normal saline.

## Data Availability

Not applicable.
